# Distinct profiles of brain atrophy in frontotemporal lobar degeneration caused by progranulin and tau mutations^☆^

**DOI:** 10.1016/j.neuroimage.2009.12.088

**Published:** 2010-11-15

**Authors:** Jonathan D. Rohrer, Gerard R. Ridgway, Marc Modat, Sebastien Ourselin, Simon Mead, Nick C. Fox, Martin N. Rossor, Jason D. Warren

**Affiliations:** a Dementia Research Centre, UCL Institute of Neurology, University College London, Queen Square, London, WC1N 3BG, UK; b Centre for Medical Image Computing, University College London, Gower Street, London, WC1E 6BT, UK

**Keywords:** Frontotemporal dementia, Frontotemporal lobar degeneration, Progranulin, Tau

## Abstract

Neural network breakdown is a key issue in neurodegenerative disease, but the mechanisms are poorly understood. Here we investigated patterns of brain atrophy produced by defined molecular lesions in the two common forms of genetically mediated frontotemporal lobar degeneration (FTLD). Nine patients with progranulin (GRN) mutations and eleven patients with microtubule-associated protein tau (MAPT) mutations had T1 MR brain imaging. Brain volumetry and grey and white matter voxel-based morphometry (VBM) were used to assess patterns of cross-sectional atrophy in the two groups. In a subset of patients with longitudinal MRI rates of whole-brain atrophy were derived using the brain-boundary-shift integral and a VBM-like analysis of voxel-wise longitudinal volume change was performed. The GRN mutation group showed asymmetrical atrophy whereas the MAPT group showed symmetrical atrophy. Brain volumes were smaller in the GRN group with a faster rate of whole-brain atrophy. VBM delineated a common anterior cingulate–prefrontal–insular pattern of atrophy in both disease groups. Additional disease-specific profiles of grey and white matter loss were identified on both cross-sectional and longitudinal imaging: GRN mutations were associated with asymmetrical inferior frontal, temporal and inferior parietal lobe grey matter atrophy and involvement of long intrahemispheric association white matter tracts, whereas MAPT mutations were associated with symmetrical anteromedial temporal lobe and orbitofrontal grey matter atrophy and fornix involvement. The findings suggest that the effects of GRN and MAPT mutations are expressed in partly overlapping but distinct anatomical networks that link specific molecular dysfunction with clinical phenotype.

## Introduction

The role of neural networks in neurodegenerative disease has attracted much recent interest (Seeley et al. 2006; Seeley et al., 2007; Seeley, 2008; Seeley et al., 2008; Borroni et al., 2008; Seeley et al., 2009) however the mechanisms of neural network breakdown remain poorly understood. Frontotemporal lobar degeneration (FTLD) presents a useful disease model in which to investigate this issue. FTLD is the second most common degenerative cause of young-onset dementia (Pickering-Brown, 2007) and constitutes a clinically, pathologically and genetically diverse group of diseases which produce anatomically restricted patterns of frontal and/or temporal lobe atrophy. Furthermore, in around a third of patients there is an autosomal dominant family history, offering the prospect of charting anatomical changes attributable to a defined molecular change (a disease-causing mutation) in the living brain. In recent years mutations in two genes, microtubule-associated protein tau (MAPT) and progranulin (GRN) have been shown to account for many familial cases of FTLD (Pickering-Brown, 2007; van Swieten et al., 2008). While the clinical spectrum of both MAPT and GRN mutations is heterogeneous, certain features occur more frequently in association with a particular molecular substrate. Patients with MAPT mutations commonly present with behavioural variant frontotemporal dementia (bvFTD) which may be accompanied by a corticobasal syndrome (CBS) or more rarely a progressive supranuclear palsy (PSP) syndrome (van Swieten & Spillantini, 2007). Cognitively, executive dysfunction is widely recognized but patients commonly develop semantic impairment later in the disease (Pickering-Brown et al., 2008) as well as prominent episodic memory difficulties (van Swieten et al., 2007). Patients with GRN mutations also present most commonly with bvFTD and there may be an associated CBS (van Swieten et al., 2008). However, unlike MAPT mutations patients in this group frequently present with primary progressive aphasia (PPA) and GRN mutations are more likely to cause early parietal lobe impairment (van Swieten et al., 2008; Pickering-Brown et al., 2008; Beck et al., 2008). Group and single case studies suggest that atrophy in association with GRN mutations is asymmetrical and widespread within the affected hemisphere, involving the frontal, temporal and parietal lobes even in the presymptomatic phase, whilst atrophy in association with MAPT mutations is more symmetrical and relatively restricted within the frontal and temporal lobes (Whitwell et al., 2007; Beck et al., 2008; Rohrer et al., 2008; Whitwell et al., 2009a).

Despite the considerable neurobiological as well as clinical interest in delineating signatures of structural damage associated with FTLD, both to further understanding of regional vulnerability in this group of disorders and to test the hypothesis that the phenomenology of FTLD syndromes reflects specific neural network dysfunction (Seeley et al., 2008; Seeley 2008; Seeley et al., 2009), there are few comparative cross-sectional imaging studies of genetic FTLD (Ghetti et al., 2008; Whitwell et al., 2009a) and little is known about the longitudinal changes in patterns of atrophy. Here we investigated the cross-sectional and longitudinal imaging features of GRN- and MAPT-associated FTLD using complementary structural imaging techniques. A particular aim of the study was to capture mutation-associated changes both in cortical regions and in underlying white matter tracts that link cortical regions within distributed neural networks.

## Materials and methods

### Subjects

The research database of patients with FTLD attending the Specialist Cognitive Disorders Clinic of the National Hospital of Neurology and Neurosurgery, London, UK, between 1992 and 2008 was retrospectively reviewed and all patients with MAPT or GRN mutations were identified. Eleven patients with a MAPT mutation (mean (standard deviation) age at baseline scan 53.5 (5.2)) and nine patients with a GRN mutation (mean (standard deviation) age at baseline scan 62.9 (6.1)) were included in the study: the MAPT mutation group comprised 8 patients with a 10 + 16 mutation and single patients with 10 + 14, S320F and G389R mutations; the GRN mutation group (described in Beck et al. (2008)) comprised four patients with a C31fs mutation, two patients with a Q130fs mutation and single patients with A199V, S203fs and E498fs mutations. Demographic and baseline neuropsychological data are shown in Table 1. There was no significant difference in mean symptom duration between the two groups, however the mean age of the MAPT group was significantly younger than the GRN group at the time of the baseline scan. All patients with MAPT mutations presented initially with a bvFTD syndrome whilst patients with GRN mutations presented with bvFTD, PPA or CBS. A control group of fifteen cognitively-normal controls (10 males, 5 females) were used for comparison (mean (standard deviation) age at baseline scan 57.5 (5.3)). All patients included in the study had at least one volumetric brain MRI scan; six patients with a MAPT mutation and four patients with a GRN mutation had two scans with no significant differences in the mean inter-scan interval between the two groups (GRN — 1.3 (0.2) years; MAPT — 2.1 (1.0) years).

### Brain imaging

All patients had MR imaging on a 1.5 T GE Signa scanner (General Electric, Milwaukee, WI). T1-weighted volumetric brain images were obtained using an IR-prepared fast SPGR sequence (TE = 5 ms, TR = 12 ms, and TI = 650 ms) with a 24-cm field of view and 256 × 256 matrix to provide 124 contiguous 1.5-mm-thick slices in the coronal plane. We initially calculated whole-brain and hemisphere volumes in all patients. The brain region was created by segmenting the whole brain using a semi-automated technique in the MIDAS software package (Freeborough et al., 1997a). For rates of atrophy in patients with longitudinal imaging, serial scans were co-registered and volume change was calculated directly using the boundary shift integral (BSI) (Freeborough et al., 1997b). BSI-derived whole-brain volume changes (BBSI) were expressed as annualized percentage change from the baseline brain volume. Analysis of hemispheric volume was also performed: scans and associated whole-brain regions were initially transformed into standard space by registration to the Montreal Neurological Institute (MNI) Template. The left and right hemispheric regions were defined using the MNI average brain which was split by dividing the whole volume along a line coincident with the interhemispheric fissure. An intersection of each individual's brain region and the hemispheric regions defined on the MNI template was generated to provide a measure of brain volume in left and right hemispheres. A left/right asymmetry ratio measure was calculated by dividing the left hemisphere volume by the right hemisphere volume. Cross-sectional and longitudinal volumetric data were analysed statistically using STATA 10.0 (Stata Corporation, College Station, TX) by examining contrasts between the group means using a linear regression model. 95% bias-corrected bootstrap confidence intervals with 1000 replicates were used.

Voxel-based morphometry (VBM) was performed on the baseline images using SPM5 software (http://www.fil.ion.ucl.ac.uk/spm) with default settings for all parameters. The images underwent an initial segmentation process in SPM5 which simultaneously estimated transformation parameters for warping grey matter (GM), white matter (WM) and cerebrospinal fluid (CSF) tissue probability maps (TPMs) onto the images. The native space GM and WM segments were then rigidly spatially normalised, using just the rotations and translations from the inverse of the TPM transformation, and resampled to 1.5 mm isotropic resolution. These “imported” images were then iteratively warped to an evolving estimate of their group-wise GM and WM average template using the DARTEL toolbox (Ashburner, 2007; Ashburner et al., 2009). The GM and WM segmentations were then normalised using the final DARTEL transformations and modulated to account for volume changes. Finally, the images were smoothed using a 6 mm full-width at half-maximum (FWHM) Gaussian kernel. Linear regression models were used to examine differences in GM and WM volume between the groups. Voxel intensity, V, was modelled as a function of group, and subject age and total intracranial volume (TIV) were included as nuisance covariates. Separate analyses were performed on the grey matter and white matter segments. Maps showing statistically significant differences between the groups were generated, correcting for multiple comparisons in the disease group-control comparisons by thresholding the images of *t*-statistics to control the False Discovery Rate (FDR) at a 0.001 significance level. For disease group comparisons maps were generated uncorrected at a 0.001 significance level. Statistical parametric maps were displayed as overlays on a study-specific template, created by warping all native space whole-brain images to the final DARTEL template and calculating the average of the warped brain images. In order to visualise hemispheric asymmetries, we performed two VBM analyses: firstly with all images in their native space; and secondly, with native images flipped in the midsagittal plane within SPM5, such that the most severely affected cerebral hemisphere was on the same side in each patient. An image was selected for flipping if it had a hemispheric asymmetry index outside the control range and more severe right hemisphere atrophy (i.e., images were flipped such that any asymmetric atrophy was displayed on the left): four images from the GRN group and three from the MAPT group met criteria for flipping.

For those patients with longitudinal imaging (6 MAPT patients and 4 GRN patients) we performed non-linear registration of each follow-up image to its corresponding baseline using a multi-scale viscous fluid algorithm (Crum et al., 2003) with a Normalised Mutual Information objective function (Crum et al., 2005). Image pairs were first flipped such that the most severely affected cerebral hemisphere was on the same side in each patient as with the VBM analysis. We computed maps of the fluid registration Jacobian determinants, encoding the relative volume change from baseline to follow-up, which can be analysed using SPM, following Scahill et al. (2002). These ‘Voxel Compression Maps’ (VCMs) were log-transformed as in Scahill et al., but instead of separating out expansion and contraction before smoothing, we separated each subject's VCM into GM, WM and CSF components according to which tissue segment had the highest probability at each voxel. The sets of tissue-specific VCMs were then spatially normalised using the DARTEL transformations and smoothed using a 6 mm FWHM Gaussian kernel as used for the VBM analysis. The GM and WM analysis masks from the cross-sectional analysis were reused, as was the average template for overlaying results. Voxel-wise statistical analysis was then performed using SPM5, with a linear regression model comprising group indicator variables and nuisance covariates of age, TIV, and inter-scan interval.

## Results and discussion

### Results

#### Volumetric data

##### Whole-brain volumes and rate of atrophy

**Fig. 1:**
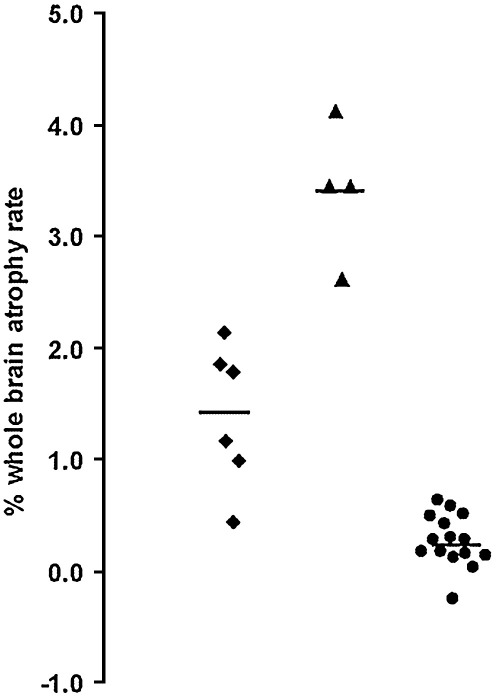
Annualized rates of whole-brain atrophy (as measured using the boundary shift integral) in the MAPT mutation (diamonds) and GRN mutation (triangles) groups as well as the controls (circles).

Baseline brain volumes were smaller in both disease groups compared to healthy controls and mean brain volume was significantly smaller in the GRN group compared to the MAPT group (Table 2). Rates of whole-brain atrophy were significantly greater in the GRN group with no overlap with the MAPT group (Table 2, Fig. 1).

##### Hemisphere volumes

**Fig. 2:**
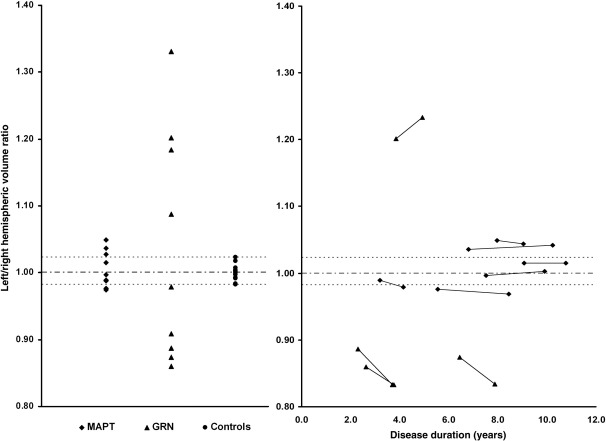
Left/right hemisphere volume ratio in the three groups (A) and in patients with longitudinal imaging as a function of disease duration i.e. time from symptom onset (B).

Baseline mean left and right hemisphere volumes were smaller in the disease groups than the controls and mean left hemisphere volume was significantly smaller in the GRN group compared to the MAPT group, with a trend to smaller mean right hemisphere volume in the GRN group (*p* = 0.07) (Table 2, Fig. 2A). The overall mean left/right asymmetry ratio was similar in all three groups, however individual cases in the GRN group were highly asymmetrical with all cases falling outside of the control range (Fig. 2A), whereas individual cases in the MAPT group were most often symmetrical with a few cases just falling outside the control range. Only a single GRN patient (GRN5) fell within the range of values of the MAPT group. Furthermore, in the patients with longitudinal imaging, GRN cases became more asymmetric as the disease progressed whilst MAPT patients remained similarly symmetrical (Fig. 2B).

#### VBM data

##### Grey matter atrophy in disease groups versus controls

**Fig. 3:**
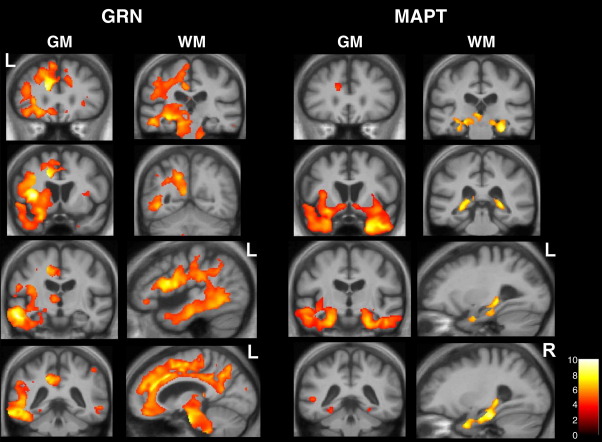
VBM analysis on grey matter (GM) and white matter (WM) regions in GRN- and MAPT-associated FTLD relative to healthy controls. Statistical parametric maps (SPMs) have been thresholded at *p* < 0.001 after false discovery rate correction over the whole-brain volume and rendered on a study-specific average group T1-weighted MRI template image in DARTEL space. The colour bar (lower right) indicates the *t* score. Left (L) and right (R) markers are shown for ease of reference however this analysis was performed on flipped images (see text). GM panels show the same series of coronal MR sections in the GRN and MAPT cases; WM panels show coronal (above) and sagittal (below) sections based on zones of maximal white matter loss in each disease group.

Patterns of grey matter atrophy differed in the MAPT and GRN groups compared to healthy controls. The GRN group analysis on unflipped images showed an overall pattern of symmetrical brain atrophy including frontal, temporal and parietal lobes, cingulate cortex and thalamus. However, this result obscures any asymmetries in favour of left or right hemisphere at individual subject level: after flipping of images so that all patients had the most affected hemisphere in the same orientation the true asymmetry of GRN disease was apparent (Fig. 3). The most significant areas of grey matter atrophy were in the inferior frontal lobe, dorsal insula, superior and middle temporal gyri, dorsal anterior cingulate cortex, precuneus and inferior parietal lobe. In contrast, the MAPT group analysis on flipped images revealed a distinct symmetrical pattern of atrophy including anterior and medial temporal lobes, orbitofrontal cortex and ventral insula with less involvement of the anterior cingulate (Fig. 3).

##### White matter atrophy in disease groups versus controls

In the GRN group compared to controls, the white matter VBM analysis showed most significant involvement of intrahemispheric long association tracts including inferior longitudinal fasciculus, superior longitudinal fasciculus, inferior fronto-occipital fasciculus and cingulum. There was additional involvement of the corpus callosum and brainstem tracts, particularly in the pons (Fig. 3). In the MAPT group compared to controls, the most significant areas of white matter loss lay in the fornices bilaterally with less marked involvement of the uncinate fasciculus (Fig. 3).

##### GRN versus MAPT group comparisons

**Fig. 4:**
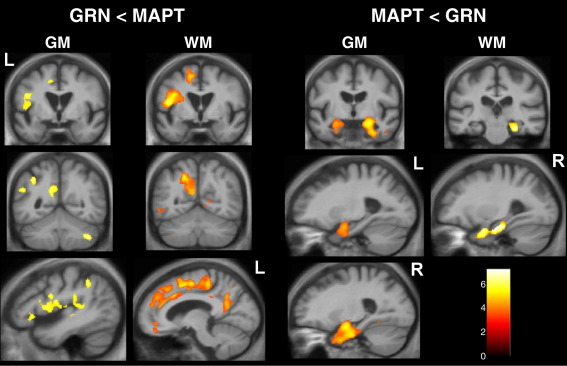
VBM analysis comparing grey matter (GM) and white matter (WM) atrophy between GRN- and MAPT-associated FTLD groups. Left-hand panels show regions where tissue intensity was reduced in the GRN group relative to the MAPT group (GRN<MAPT) and right-hand panels show regions where tissue intensity was reduced in the MAPT group relative to the GRN group (MAPT<GRN). Statistical parametric maps (SPMs) have been thresholded at *p* < 0.001 (uncorrected) and rendered on the same template used in Fig. 3. The colour bar (lower right) indicates the *t* score. Left (L) and right (R) markers are shown for ease of reference however this analysis was performed on flipped images (see text). Sections shown are based on zones of maximal grey and white matter loss in each disease group.

Comparing the two mutation groups directly in the flipped VBM analysis (Fig. 4) revealed mutation-associated atrophy profiles involving brain regions previously identified in the control comparison. The GRN group had more marked and asymmetric grey matter loss in inferior frontal lobe, dorsal insula, posterior temporal and inferior parietal lobes and precuneus, and more marked and similarly asymmetric white matter loss in superior longitudinal fasciculus and cingulum. The MAPT group had more marked grey matter loss in the anterior and medial temporal lobes bilaterally and more prominent involvement of the fornices (Fig. 4).

#### Longitudinal non-linear registration data

**Fig. 5:**
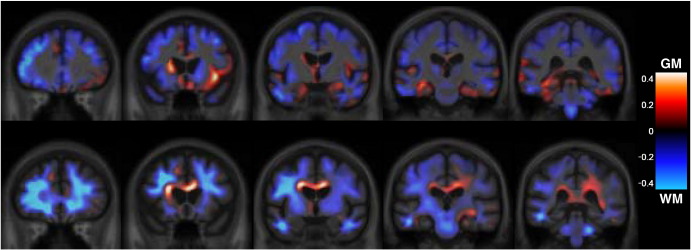
Longitudinal voxel compression map analysis showing comparison between GRN- and MAPT-associated FTLD groups. Red indicates more severe longitudinal atrophy in the MAPT group, and blue greater in GRN. Top panel shows grey matter (GM) maps and bottom panel shows white matter (WM) maps, which are overlaid on the same average template used in Fig. 3. Because of the very small groups, we present only unthresholded maps of the group-difference, rather than thresholded *t*-maps, which show only a few isolated peaks at an uncorrected 0.001 level. These unthresholded maps of effect–size show the character, but not the significance, of the group differences.

Differential patterns of longitudinal volume loss in the two mutation groups were similar to those seen in the cross-sectional VBM analysis (Fig. 5). The GRN group showed longitudinal volume loss asymmetrically involving mainly the inferior frontal, superior temporal, and inferior parietal lobes, precuneus and cingulate cortex as well as the long association white matter tracts. The MAPT group showed longitudinal volume loss symmetrically involving the anteromedial temporal lobes, orbitofrontal cortex and white matter tracts including the corpus callosum.

## Discussion

Using convergent imaging techniques, we have shown that GRN and MAPT mutations are associated with distinct profiles of neuronal loss affecting distributed cortical areas and their white matter connections. GRN mutations are associated with asymmetric atrophy of frontal, insular, cingulate, parietal and temporal areas linked by intrahemispheric long association tracts, while MAPT mutations are associated with more restricted but bi-hemispheric atrophy of anteromedial temporal and orbitofrontal areas linked via the fornices and uncinate fasciculi. Disease evolution is more rapid in GRN- than MAPT-associated FTLD, as predicted from the more distributed cerebral damage associated with GRN mutations. Moreover, the degree of asymmetry increases over time in the GRN- (but not the MAPT) associated cases: in conjunction with the evidence presented here for asymmetric longitudinal intrahemispheric volume loss, this increasing asymmetry implies that the pathological process in GRN-associated FTLD is focused within the maximally affected hemisphere. The profiles of grey matter involvement demonstrated here help to integrate previous evidence concerning anatomical signatures and disease evolution in GRN and MAPT mutation cases. In particular, our findings suggest an anatomical substrate for the shorter disease duration (Beck et al., 2008) and greater asymmetry of disease involvement (Beck et al., 2008; Ghetti et al., 2008; Rohrer et al., 2008) observed with GRN compared with MAPT mutations while underlining the individual variability in lateralisation that may make asymmetry more difficult to detect in group morphometric studies (Whitwell et al., 2009a).

White matter involvement in FTLD has been little studied but long association tracts including the anterior cingulum and superior longitudinal fasciculus have been implicated (Seeley et al., 2008; Borroni et al., 2008). A diffusion tensor imaging study in presymptomatic patients with GRN mutations showed left uncinate fasciculus, arcuate fasciculus (part of the superior longitudinal fasciculus), and left inferior fronto-occipital fasciculus (Borroni et al., 2008). In the present study, the use of an unbiased technique (VBM) has revealed distinct patterns of white matter tract involvement associated with GRN and MAPT mutations, including pathways (such as the fornix) not previously identified in imaging studies of genetic FTLD. It will be important in future studies of genetic FTLD to investigate the complementary information about white matter tracts that can be provided by diffusion tractography (Mori & Zhang, 2006).

Neural network dysfunction has been proposed to underpin phenotypic features of neurodegenerative disease including FTLD (Seeley et al., 2009), and in particular, behavioural dysfunction in bvFTD has been ascribed to selective vulnerability of von Economo neurons within a frontal–insula–anterior cingulate network (Seeley et al., 2006; Seeley et al., 2007; Seeley 2008; Seeley et al., 2008). The present findings indicate that this network is affected by both GRN and MAPT mutations, suggesting that it is vulnerable to different pathological processes in FTLD. However, the most significant areas of atrophy here were differentiated according to the underlying molecular abnormality: the atrophy profile of MAPT mutations is consistent with involvement of a ventral orbitofrontal–medial temporal–ventral insula network while the atrophy profile of GRN mutations is consistent with involvement of a more dorsal and asymmetrical anterior cingulate–dorsal insula–temporal–parietal network. This suggests that large-scale neural network dysfunction may be a signature of specific molecular pathologies within the FTLD spectrum. The genetically defined networks identified here are aligned with anatomically similar functional networks delineated in functional connectivity and resting-state network fMRI studies of healthy individuals (Beckmann et al., 2005; Damoiseaux et al., 2006; Margulies et al., 2007; Seeley et al., 2009). Network dysfunction here may provide a neuropathological bridge between molecular dysfunction and clinical phenotype in different genetically mediated forms of FTLD: clinically, MAPT mutations produce behavioural symptoms (especially disinhibition) and later semantic impairment consistent with involvement of the ventral behavioural–semantic network (Seeley et al., 2009); while GRN mutations may produce bvFTD (with early involvement of the dorsal network in the right cerebral hemisphere), progressive aphasia (with early involvement of the dorsal network in the left cerebral hemisphere) or a corticobasal syndrome (with early involvement of more posterior parts of the dorsal network in either hemisphere).

A crucial unsolved question concerns the mechanisms whereby different molecular lesions may produce strikingly dissimilar patterns of neural network breakdown. There are three interrelated pathophysiological issues here: firstly, how one mutation produces asymmetrical cerebral damage and another more symmetrical damage; secondly, how these distinctive patterns of atrophy are maintained or amplified as the disease evolves; and finally, how phenotypic variation arises such that a particular mutation may selectively damage different cerebral hemispheres even between members of the same family (Beck et al., 2008). The variable clinical presentation of genetic FTLD suggests that molecular lesions do not specify a precise initial anatomical locus of brain damage: the initiation of disease in a particular brain region may be a stochastic event or could reflect hemispheric vulnerability due to developmental or other environmental factors (Mesulam, 2009). However, the evidence from this study suggests that, once initiated, the pattern of disease evolution and the type of evolution that can occur are constrained by the underlying molecular abnormality. Particular mutations are likely to exert their effects via the patterns of large-scale network connectivity that exist in the healthy brain, with connectivity between homotopic cortical areas (which is variable in different parts of the brain) being a crucial factor in linking a particular molecular lesion with symmetrical or asymmetrical network involvement (Stark et al., 2008). At a molecular level, GRN and MAPT are likely to be differently toxic to neurons: loss of GRN-mediated trophic support (Eriksen & Mackenzie, 2008) might particularly disrupt long axonal connections within a hemisphere, whereas in MAPT-associated FTLD, toxic gain of function (Gendron & Petrucelli, 2009) and the effects of diffusible tau with local spread to neighbouring brain regions (Brunden et al., 2008; Clavaguera et al., 2009) might lead to relatively restricted damage maximally affecting nearby synapses and local interneuronal populations within a functional brain region.

The concept of large-scale neural network breakdown linked to specific molecular lesions may be relevant to the pathogenenesis of a number of neurodegenerative pathologies. Specific mutations within a general category of genetically mediated neurodegeneration (such as GRN or MAPT-associated FTLD) may mediate more fine-grained profiles of brain damage, as suggested by recent evidence within the MAPT spectrum (Whitwell et al., 2009b). The small case numbers in the present study do not allow conclusions to be drawn about such fine-grained mutation specificity, underlining a need for larger mutation cohorts recruited via multiple centres in future studies. A related priority is the identification of earliest network-level changes in presymptomatic mutation carriers. In particular, further work is required to test the hypotheses proposed here and to establish the true status of molecular network dysfunction in the pathogenesis of FTLD and indeed, the broader spectrum of neurodegenerative disease.

## Figures and Tables

**Table 1:** Demographic, genetic and neuropsychological data for the patient groups.

Patient	Presenting syndrome	Mutation	Gender	Age at scan	Symptom duration at scan	VIQ	PIQ	RMT words	RMT faces	GNT	VOSP	Executive dysfunction
MAPT1	bvFTD	10 + 16	M	54.5	7.5	91	96	< 5th	< 5th	< 5th	< 5th	Pass
MAPT2	bvFTD	10 + 16	M	58.0	8.0	97	107	< 5th	< 5th	< 5th	> 50th	Pass
MAPT3	bvFTD	S320F	M	58.7	7.7	107	128	> 75th	5–10th	< 5th	25–50th	Pass
MAPT4	bvFTD	G389R	M	46.1	3.1	77	70	< 5th	< 5th	< 5th	> 10th	Fail
MAPT5	bvFTD	10 + 16	F	48.8	6.8	83	81	10–25th	< 5th	< 5th	50–75th	Pass
MAPT6	bvFTD	10 + 16	F	53.2	3.2	90	84	10–25th	< 5th	< 5th	25–50th	Fail
MAPT7	bvFTD	10 + 14	M	50.1	9.1	100	96	< 5th	< 5th	< 5th	NT	NT
MAPT8	bvFTD	10 + 16	M	52.5	5.5	104	121	5–10th	5–10th	< 5th	> 75th	Pass
MAPT9	bvFTD	10 + 16	M	45.9	8.9	99	93	< 5th	< 5th	5–10th	> 5th	Pass
MAPT10	bvFTD	10 + 16	M	60.7	2.7	99	105	< 5th	< 5th	< 5th	> 10th	Pass
MAPT11	bvFTD	10 + 16	F	56.8	1.8	85	97	< 5th	25–50th	50–75th	> 5th	Pass
GRN1	bvFTD	C31fs	M	67.4	1.4	84	72	< 5th	< 5th	10–25th	< 5th	Fail
GRN2	bvFTD	Q130fs	F	65.5	3.5	59	74	< 5th	< 5th	< 5th	< 5th	Fail
GRN3	PPA	C31fs	F	68.3	2.3	85	108	50–75th	10–25th	< 5th	25–50th	Pass
GRN4	bvFTD	Q130fs	M	65.9	3.9	107	95	25th	10–25th	> 75th	> 5th	Fail
GRN5	PPA	C31fs	F	63.0	5.0	84	86	10–25th	< 5th	25–50th	25–50th	Fail
GRN6	PPA	S203fs	M	50.6	2.6	66	98	25–50th	> 75th	< 5th	> 50th	Pass
GRN7	bvFTD	C31fs	M	56.4	3.4	88	80	25–50th	25–50th	50–75th	> 50th	Fail
GRN8	PPA/CBS	E498fs	F	68.4	6.4	Unable	69	5–10th	< 5th	< 5th	< 5th	NT
GRN9	CBS	A199V	M	60.7	5.7	61	Unable	Unable	Unable	< 5th	< 5th	Fail

Verbal IQ (VIQ) and Performance (PIQ) scores are taken from the WAIS-R. Recognition Memory Test (RMT) for Words and Faces, Graded Naming Test (GNT) and Visual Object and Space Perception (VOSP) battery results are quoted in percentile scores where a score below the 5th percentile is considered impaired. Executive function tasks are the Weigl or Wisconsin Modified Card Sorting Tasks or the Stroop task and are quoted as pass or fail.

**Table 2:** Volumetric cross-sectional and longitudinal data.

Mean (95% confidence intervals)	*MAPT* mutations	*GRN* mutations	Controls
Whole-brain volume (ml)	1117.3 (1079.6, 1162.5)^a^	996.8 (914.0, 1099.2)^a^^,^^b^	1230.5 (1180.2, 1272.6)
Whole-brain BSI atrophy rate (%/yr)	1.4 (0.9, 1.9)^a^	3.4 (2.8, 4.0)^a^^,^^b^	0.3 (0.1, 0.4)
Left hemisphere volume (ml)	553.5 (536.4, 573.4)^a^	496.4 (460.8, 545.4)^a^^,^^b^	605.2 (581.3, 626.3)
Right hemisphere volume (ml)	552.0 (531.8, 574.9)^a^	489.8 (437.0, 562.1)^a^	605.9 (581.6, 625.3)
Left/right hemisphere ratio	1.00 (0.99, 1.02)	1.03 (0.93, 1.15)	1.00 (0.99, 1.01)

a *p* < 0.05 disease group significantly worse than Controls.

b *p* < 0.05 *GRN* mutation group significantly worse than *MAPT* mutation group.
